# Tunable Wetting Property in Growth Mode-Controlled WS_2_ Thin Films

**DOI:** 10.1186/s11671-017-2030-z

**Published:** 2017-04-07

**Authors:** Byoung Ki Choi, In Hak Lee, Jiho Kim, Young Jun Chang

**Affiliations:** Department of Physics, University of Seoul, Seoul, 02504 Republic of Korea

**Keywords:** WS_2_, Wetting property, Morphology control, Chemical vapor deposition, Raman spectroscopy, Transmission electron microscopy

## Abstract

We report on a thickness-dependent wetting property of WS_2_/Al_2_O_3_ and WS_2_/SiO_2_/Si structures. We prepared WS_2_ films with gradient thickness by annealing thickness-controlled WO_3_ films at 800 °C in sulfur atmosphere. Raman spectroscopy measurements showed step-like variation in the thickness of WS_2_ over substrates several centimeters in dimension. On fresh surfaces, we observed a significant change in the water contact angle depending on film thickness and substrate. Transmission electron microscopy analysis showed that differences in the surface roughness of WS_2_ films can account for the contrasting wetting properties between WS_2_/Al_2_O_3_ and WS_2_/SiO_2_/Si. The thickness dependence of water contact angle persisted for longer than 2 weeks, which demonstrates the stability of these wetting properties when exposed to air contamination.

## Background

Layered transition metal dichalcogenides (TMDs), such as *MX*_2_ (*M* = Mo or W, *X* = S, Se, or Te), have intriguing transition behaviors in electronic structures and optical properties, subject to layer thickness. TMDs have been intensively explored for diverse applications, such as transparent flexible devices [1–8], valleytronics [9, 10], and optoelectronics [11–15]. Other important aspects of TMDs are their tribological properties, such as friction and wetting characteristics. By understanding the tribological aspects, one may control liquid-solid interface in nanoscale sensors [16] and make self-cleaning surfaces on transparent electronic devices [17, 18]. Such applications, especially, demand air exposure stability of the film surfaces, because the surfaces should be exposed to liquid-solid interaction either for attracting or repelling liquid droplets. Among the layered materials, WS_2_ powder lubricant is known to be useful in harsh conditions due to its high chemical stability compared to graphite and MoS_2_ powders [19–21]_._

Depending on the layer thickness, both graphene and TMDs show interesting wetting characteristics. To date, wetting has been understood in terms of several important parameters: wettability of supporting substrate, crystallinity or morphology of a TMD film, and airborne contaminants. Recently, Chow et al. suggested a model to understand the influence of these parameters as a function of two major types of interactions: short-range van der Waals interactions and long-range dipole-dipole interactions [22]. Based on this model, one could understand that flat, uniform TMD layers on SiO_2_/Si substrates show a strong thickness dependence of the water contact angle (CA). This thickness dependence becomes weakened after aging in air, and such aging effect is saturated after 7 days [22]. However, more drastic changes in wetting behavior could possibly be achieved by combining both wetting translucency and artificial nanoscale patterning. Such nanoscale patterning of the TMD layer could be realized by using photolithography and alternatively also by controlling the initial growth mode to obtain nanoscale islands [23].

Chemical vapor deposition (CVD) is a robust method for fabricating large-area TMD thin films and has the advantages of being low cost, scalable, and industry compatible [4, 24–26]. TMD thin films can be synthesized either by evaporating metal-oxide powder or by converting pre-deposited metal or metal-oxide films in the presence of chalcogen atmosphere. In particular, displacing the oxygen in pre-deposited metal-oxide films in chalcogen atmosphere has the advantage of obtaining continuous films with controlled film thickness. Moreover, controlling growth parameters, such as temperature, thickness, and substrate, can lead to a variety of film morphologies and crystallinity [4, 24, 27].

In this paper, we report on the synthesis of WS_2_ films where different surface morphologies were obtained by employing different substrates (SiO_2_/Si and Al_2_O_3_). By adopting a step-like wedge growth method, we tested the influence of film thickness and underlying substrate on the behavior of the water CA. We find that the crystallinity of the substrate determines surface roughness and the continuity of the as-grown films, which strongly affects the interaction of water with the ultrathin films and underlying substrates. We also discuss the influence of airborne contaminants for the stability of the water CA.

## Methods

**Fig. 1:**
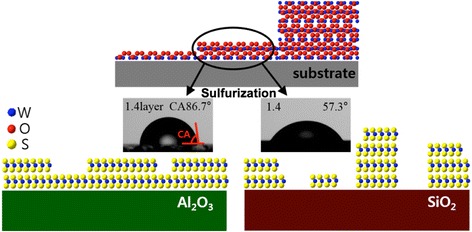
Schematic diagram of a step-like thickness gradient WO_3_ film and the sulfurized WS_2_ films on Al_2_O_3_ and SiO_2_/Si substrates. Water contact angle (CA) images show different CAs on different substrates for 1.4 monolayer of WS_2_

The WO_3_ thin films were deposited in an e-beam evaporator system (Korea Vacuum Tech) and sulfurized using CVD (Scientech). For evaporation of WO_3_, chunk pieces of WO_3_ (99.9%) were used as a source. The sapphire (Al_2_O_3_) (001) and SiO_2_/Si substrates with 300-nm-thick dry-oxidized SiO_2_ were thoroughly cleaned with acetone, ethanol, and deionized water. Before introducing the substrates into the e-beam evaporator chamber, the substrates were pre-annealed at 400 °C for 1 h in high vacuum (<5 × 10^−7^ Torr) to minimize surface adsorbents. The gradient-thickness WO_3_ films were deposited by using a manual shutter in front of the long pieces of substrate. A schematic diagram is shown in Fig. 1. WO_3_ films with step-like thickness variation were prepared with *d*_WO3_ = 0.5–10 nm. These films were then sulfurized in a CVD chamber at 800 °C for 1 h. During the whole CVD process, the total pressure was maintained at 0.5 Torr by mixing Ar and H_2_ gases with flow rates of 80 and 10 sccm, respectively. Similar sulfurization methods have been reported for fabrication of centimeter-scale WS_2_ films on SiO_2_/Si wafers and WSe_2_ films on Al_2_O_3_ wafers [24, 28–30].

The static water CA measurements were carried out on as-grown films by using a goniometer (Rame-hart, Model 200) with deionized water (>3 MΩ) at a temperature of 23 °C and 60% humidity. The CA measurements were repeated under similar conditions 20 days later to check the effect of aging due to air exposure.

The crystallinity and thickness were measured using a room temperature micro-Raman spectrometer (inVia, Renishaw). For the Raman measurement, the 514.5 nm excitation laser was first calibrated with the Si peak at 520.0 cm^−1^ as a reference and intensities were normalized to the main peak of WS_2_ at 355 cm^−1^. The film microstructure was studied using a transmission electron microscopy (TEM) (JEM-ARM200F, JEOL) with a Schottky-type field emission gun (FEG) operated at 200 keV. The cross-sectional specimens are prepared with conventional processes: cutting, gluing, polishing, and ion milling (PIPS 691, GATAN) operated at 5 keV.

## Results and Discussion

**Fig. 2:**
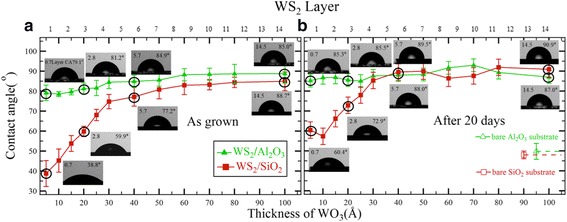
Water CA measurements of **a** the as-grown WS_2_ films on Al_2_O_3_ and SiO_2_/Si substrates and of **b** the same samples after 20 days. *Dashed lines* indicate the CA values of bare Al_2_O_3_ (*green*) and SiO_2_/Si (*red*) substrates

We found an interesting thickness dependence of the water CA values for WS_2_ films with SiO_2_/Si and Al_2_O_3_ substrates. To minimize the influence of airborne contaminants [31, 32], we measured the water CA on CVD as-grown samples within 2 h, by keeping the samples in a portable vacuum desiccator. Figure 2a shows the CA data obtained for the as-grown WS_2_/SiO_2_/Si and WS_2_/Al_2_O_3_ sample series. Both sample series showed gradual decrease of CA from *d*_WO3_ = 8 to 3 nm. Interestingly, below *d*_WO3_ = 3 nm, the CA of the WS_2_/SiO_2_/Si samples sharply decreased, while the CA of the WS_2_/Al_2_O_3_ samples decreased gradually. We note that the gradual decrease in CA was similar to observations in uniform WS_2_ films on SiO_2_/Si substrates, as reported earlier [22]. Such similarity suggested that the thickness dependence of CA may be more sensitive to the film morphology than to the underlying substrate.

**Fig. 3:**
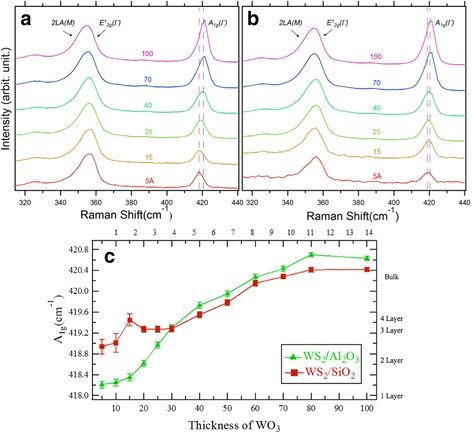
**a** Raman spectra from 0.5-nm- to 10-nm-thick WS_2_ grown on Al_2_O_3_ and **b** SiO_2_ substrate as a function of WO_3_ thickness (*d*_WO3_) (514.5 nm laser excitation, 300 K). The peaks at ~355 and ~420 cm^−1^ correspond to overlap of 2LA(M) and E^1^_2g_(Γ) peaks and A_1g_(Γ) peak, respectively. *Blue and red dashed lines* indicate the A_1g_(Γ) peak position of the thick sample (*d*_WO3_ = 10 nm) and thin sample (*d*_WO3_ = 0.5 nm), respectively. Intensities are normalized with the A_1g_ peaks. **c** Thickness dependence of the A_1g_(Γ) peak position

After the CA measurements on the as-grown samples, we carried out Raman spectroscopy measurements to analyze crystallinity and film thickness, as shown in Fig. 3. As *d*_WO3_ increases, the Raman spectrum shows a gradual change, indicating that the WS_2_ film thickness also varies with *d*_WO3_. We clearly observe blue shifting of the A_1g_ peak (~420 cm^−1^) for both WS_2_/SiO_2_/Si and WS_2_/Al_2_O_3_ sample series. Figure 3c shows *d*_WO3_ dependence of the A_1g_ peak positions, which is often used for estimating the WS_2_ layer thickness [24, 29, 33]. Both series showed a gradual decrease of the A_1g_ peak as *d*_WO3_ decreased. However, there was a significant deviation between the two sample series for *d*_WO3_ < 3 nm. The strongest peak (near 355 cm^−1^) showed a gradual change in shape, corresponding to change in the ratio of intensities of two superposed peaks, 2LA (~352 cm^−1^) and E^1^_2g_ (~356 cm^−1^).

From the Raman spectrum analysis, we identified each sample with nominal WS_2_ film thickness, assuming a uniform film. As indicated in the right axis of Fig. 3c, we estimated WS_2_ layer thickness based on both the systematic shift of the A_1g_ peak and the change in intensity ratio of 2LA and E^1^_2g_ peaks, compared with both the reported exfoliated and CVD-grown samples [24, 29, 33]. The WS_2_/Al_2_O_3_ sample series showed linear thickness variation between 2.1 and 5.7 monolayers (ML). However, the WS_2_/SiO_2_/Si sample series had significantly non-linear thickness variation for thickness ranging between 1.4 and 4.3 ML, which coincided with the range where there was significant deviation of thickness-dependent CA for very thin films, i.e., *d*_WO3_ < 3 nm.

**Fig. 4:**
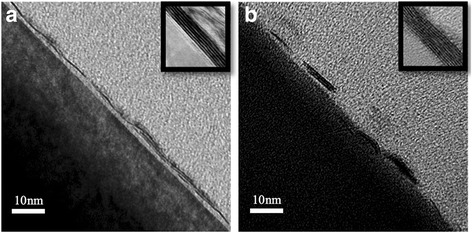
High-resolution transmission electron microscopy cross section images of WS_2_ (*d*_WO3_ = 1 nm) grown on **a** Al_2_O_3_ and **b** SiO_2_ substrates. *Insets* show corresponding image for the thick WS_2_ (*d*_WO3_ = 8 nm)

To check the film thickness and crystallinity, we performed cross section TEM analysis for both thin (*d*_WO3_ = 1.0 nm) and thick (*d*_WO3_ = 8.0 nm) samples for both substrates. Figure 4 shows the TEM images of the thin samples grown on (a) Al_2_O_3_ and (b) SiO_2_/Si substrates. The Al_2_O_3_ substrate supported a nearly continuous film with thickness between 1–3 ML, well in agreement with the nominal layer thickness of 1–2 ML, as shown in Fig. 3c. This uniform growth mode corresponds to a nearly linear shift of the A_1g_ peak as a function of *d*_WO3_. On the other hand, the film on SiO_2_/Si substrate had several patches with thicknesses of 1–4 ML and lateral width of ~10 nm. Such nanoscale patches with thickness of a few MLs were consistent with the estimated thickness of 3 ML based on the Raman spectra for *d*_WO3_ < 3 nm, as shown in Fig. 3c. Therefore, we understand that very thin WS_2_ films grow with quite different growth modes during sulfurization, as schematically illustrated in the bottom of Fig. 1.

While very thin films showed contrasting film morphologies between two different substrates, thick films (*d*_WO3_ = 8.0 nm) on both substrates showed continuous film morphologies, as shown in insets of Fig. 4. This is also consistent with the Raman spectroscopy analysis, in which the films on both substrates showed nearly the same A_1g_ peak values for thick films (see Fig. 3). By comparing the Raman spectroscopy and TEM results, we confirmed the nominal layer thicknesses of WS_2_ films, as indicated on the right axis in Fig. 3c. The relationship of WS_2_ layer thickness to *d*_WO3_ is indicated at the upper axis in Fig. 2.

Based on the differences in film morphology, we try to understand the interesting CA behavior. Previous reports about CA behavior on TMDs and graphene layers showed distinct thickness dependence between oxide and metal substrates due to differences in the charge screening of substrates. Here, we consider two models for oxide substrates: the uniform film model [22] and the growth-mode change model [31]. The uniform film model explains the gradual thickness dependence of CA for uniform TMD layers grown on SiO_2_/Si substrates. This model is consistent with our uniform WS_2_ layers on Al_2_O_3_ substrates. On the other hand, the growth-mode change model explains the larger thickness dependence of CA for rough TMD films, due to different crystal orientation and defects. This model well explains the sharper thickness dependence of CA for our rough WS_2_ films on SiO_2_/Si substrates. Therefore, we can reason that the growth-mode difference primarily affects the contrast of the change in CA for WS_2_ films on different substrates.

We note that the wetting transparency model also explains the strong thickness dependence of CA. C.-J. Shih et al. reported that 30% of the van der Waals interactions can be transmitted through the uniform ML graphene between water and the substrate [23]. In our uniform films on Al_2_O_3_ substrates, however, we observed only ~10% decrease in CA in the thinnest film compared to the thick film (~14 ML). This minute decrease of CA may be due to either the wetting transparency of uniform ML WS_2_ films or the aging process with absorption of airborne hydrocarbons [22]. However, the wetting transparency model cannot explain the strong thickness dependence of CA in the case of non-uniform films.

Furthermore, to understand the influence of airborne contaminants [32, 34], we repeated the CA measurements after keeping the samples in ambient conditions for 20 days, to exceed the air exposure saturation period of 1 week in the previous study [22]. As shown in the right-hand graphs of Fig. 2, extended air exposure diminished the overall thickness dependence of the water CA. For the WS_2_/Al_2_O_3_ samples with continuous morphology, the change in CA as a function of film thickness was smaller than the noise level. For the WS_2_/SiO_2_/Si samples with rough morphology, however, the distinct thickness dependence in CA persisted. The air exposure effect of continuous film is comparable with previous reports on the air susceptibility of continuous films with variable thickness, in which the air exposure influence is saturated after 1 week. The monolayer WS_2_, in particular, showed drastic aging behavior of CA within a few days, which made the thickness dependence of CA weak [22]. We note that further control experiment of air exposure by completion of sample preparation in the globe box is highly demanded as a future study.

However, we speculate that nanoscale crystallinity plays an important role for the air-stable CA behavior of the roughly grown films. The modified crystallinity at the nanoscale changes surface energy, which induces rather air-stable CA properties even on ultrathin film surface [29]. This robust thickness dependence of CA implies that one can selectively grow such rough morphology film to repel or attract liquid droplets, which becomes a building block of fluid control at nanoscale. Such air-stable thickness-dependent CA behavior could become important for applications, such as transparent electronic devices and microfluidic applications, where the surface tribological properties should be robust for extended exposure to atmosphere or liquid [17, 18, 31].

## Conclusions

We fabricated ultrathin WS_2_ films with very different surface morphologies on different substrates, i.e., hexagonal crystalline Al_2_O_3_ (001) and amorphous SiO_2_. WS_2_ films with different surface morphologies showed distinct differences in the thickness dependence of the water CA. The thickness-dependent CA behaviors were stable following 20 days of air exposure. These transparent WS2 films should be useful for applications with transparent electronic devices.

## Abbreviations

CA: Contact angle
CVD: Chemical vapor deposition
FEG: Field emission gun
TEM: Transmission electron microscopy
TMD: Transition metal dichalcogenide
